# New Zealand Society for the Health of Women and Children

**Published:** 1914-06

**Authors:** 


### New Zealand Society for the Health of Women and Children.

The Children's Bureau, Department of Labor, has just issued a pamphlet on baby-saving methods in New Zealand as illustrated in the work of the Society for the Health of Women and Children. Miss Julia C. Lathrop, Chief of the Bureau, in her letter transmitting the publication to Secretary Wilson, explains her reasons for issuing the pamphlet. She says:

"The infant mortality rate of New Zealand has been for some time recognized as the lowest of any country in the world, and it is stated that recent further reductions are due in large measure to the activity of this society. As an example of its value, the consul general states that the work of the society has reduced infant mortality in Dunedin, a residential city of about 60,000 inhabitants, 50 per cent during five years, from 1907 to 1912. Because of the absence of adequate birth and death registration in the United States, the infant death rate of this country as a whole is unknown, but estimates tend to show that it is at least twice the rate in New Zealand, which the registrar general of that country reported in 1912 to be 51 per 1000. New Zealand, like certain of our States, is a young and vigorous country with a scattered population and with no large cities, and there is every reason to believe that similar volunteer effort in this country would produce similar results. In view of the marked and growing interest in the preservation of infant health in the smaller cities and rural communities of the United States, I believe that the account of the methods of the New Zealand society is especially timely. It will be seen that public interest is strongly enlisted in its efforts. Seventy volunteer committees in as many districts maintain the educational and nursing work in conjunction with the central office, and the government itself assists in various ways. The detailed statement which follows is not offered for the purpose of urging exact reproduction of the New Zealand organization, but rather to stimulate interest in working out whatever methods are practicable locally for securing the same results."

CONCLUSIONS DRAWN FROM STUDY OF METHODS IN NEW ZEALAND.

1. The recognition that not only in cities but in country districts provision should be made for instructing mothers in the care of babies; teaching young girls all practical methods of home making, including baby hygiene and feeding; giving proper hospital care to sick babies; and maintaining conference where mothers can have their children examined and can thus learn of any bad condition before it has progressed beyond recovery.

2. The need of definite knowledge of just what the problem is in the different communities. This knowledge it is not possible to obtain in many districts of the United States, because, on account of the incomplete registration of births and deaths, this country does not know how many babies are born and how many die. Therefore, to urge the passage of good registration laws in States in which such laws do not exist and to force efficiency in administering the registration laws in other States is a definite requirement.

3. The need of co-operation between volunteer and public health authorities in reducing the infant mortality.

4. Recognition of the merit of the methods of the New Zealand society for consideration by club women and others in making plans for infant-welfare campaigns in rural communities in the United States.

5. The value of methods which include districting the territory in a State and organizing local committees having supervision of the welfare work; the employment of nurses whose services are chiefly educational; newspaper publicity; and the publication of pamphlets and other literature on hygiene and the care of babies and children, containing advice vouched for by the best medical authorities and expressed in direct, simple language.

